# Supplementary material for: Smilax weniae, a New Species of Smilacaceae from Limestone Areas Bordering Guizhou and Guangxi, China
Source: Plants (Basel). 2022 Apr 11;11(8):1032. doi: 10.3390/plants11081032 (PMC9028124; doi:10.3390/plants11081032)
Supplement: Supplementary file 1 [file plants-11-01032-s001.zip › plants-1606369-supplementary.pdf]

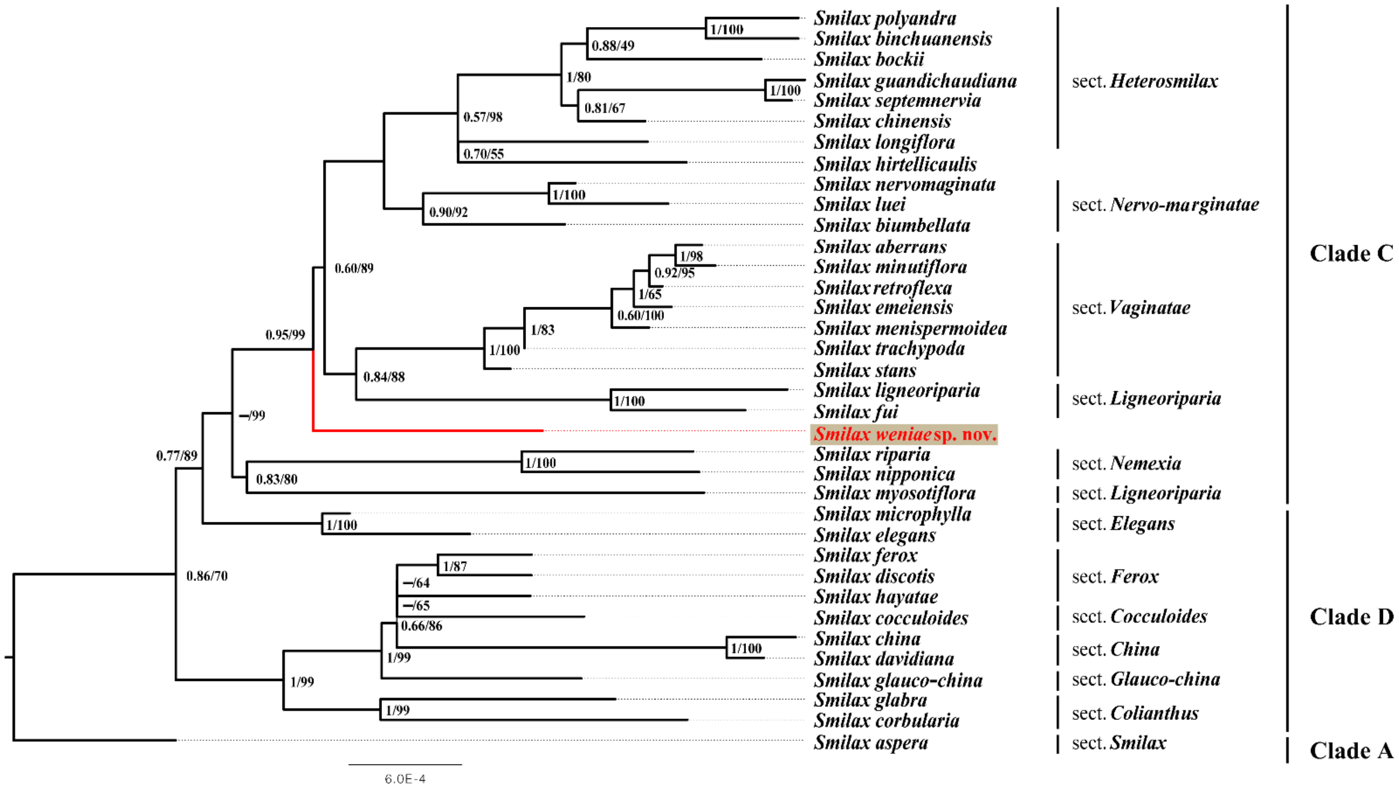

Figure S1. Maximum likelihood phylogram of the ptDNA of *Smilax weniae* and 34 other *Smilax* species, representing an Old World Smilacaceae clade. Posterior probabilities (PP > 0.50) and bootstrap values (BS > 50%) based on Bayesian and maximum likelihood (ML) analyses are shown near the branches. Clade information adpoted from Qi et al.[1].

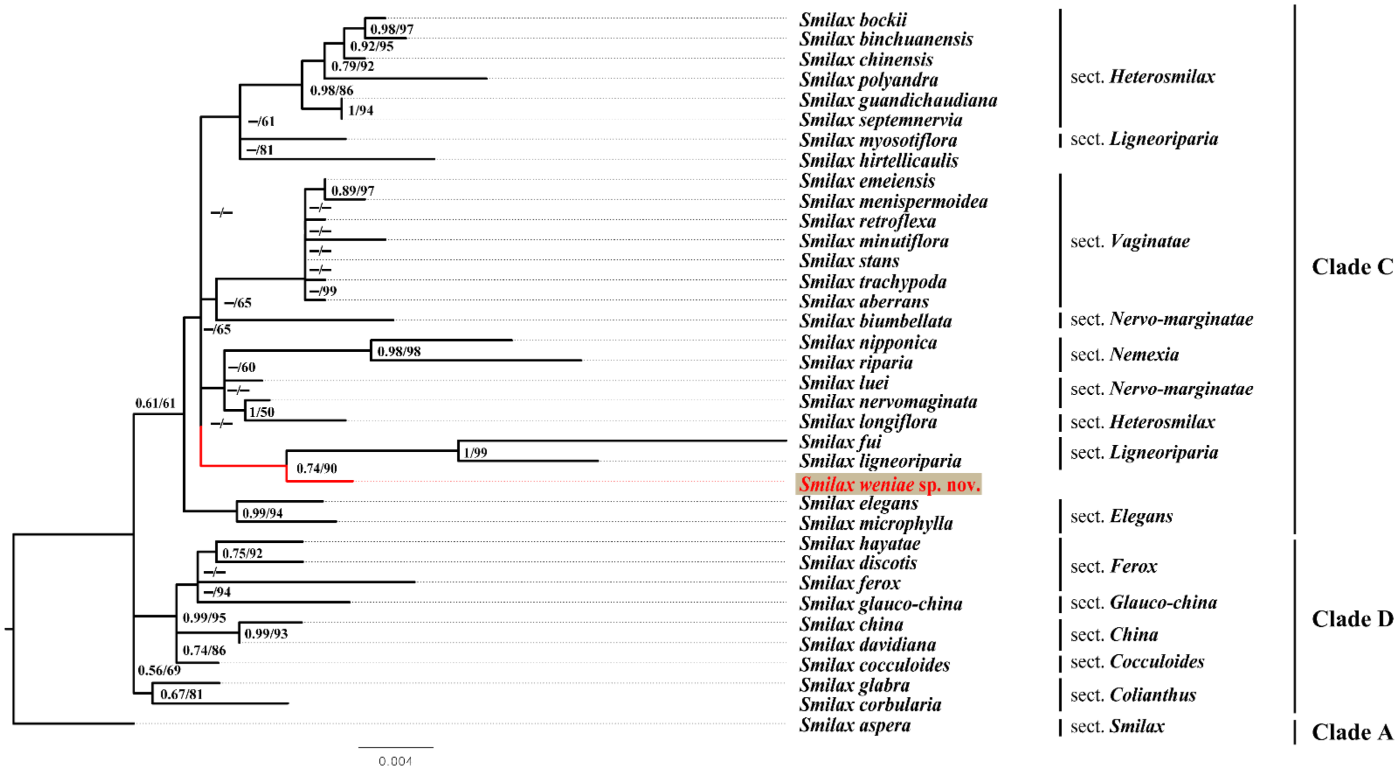

Figure S2. Maximum likelihood phylogram of the nrITS of *Smilax weniae* and 34 other *Smilax* species, representing an Old World Smilacaceae clade. Posterior probabilities (PP > 0.50) and bootstrap values (BS > 50%) based on Bayesian and maximum likelihood (ML) analyses are shown near the branches. Clade information adpoted from Qi et al.[1].

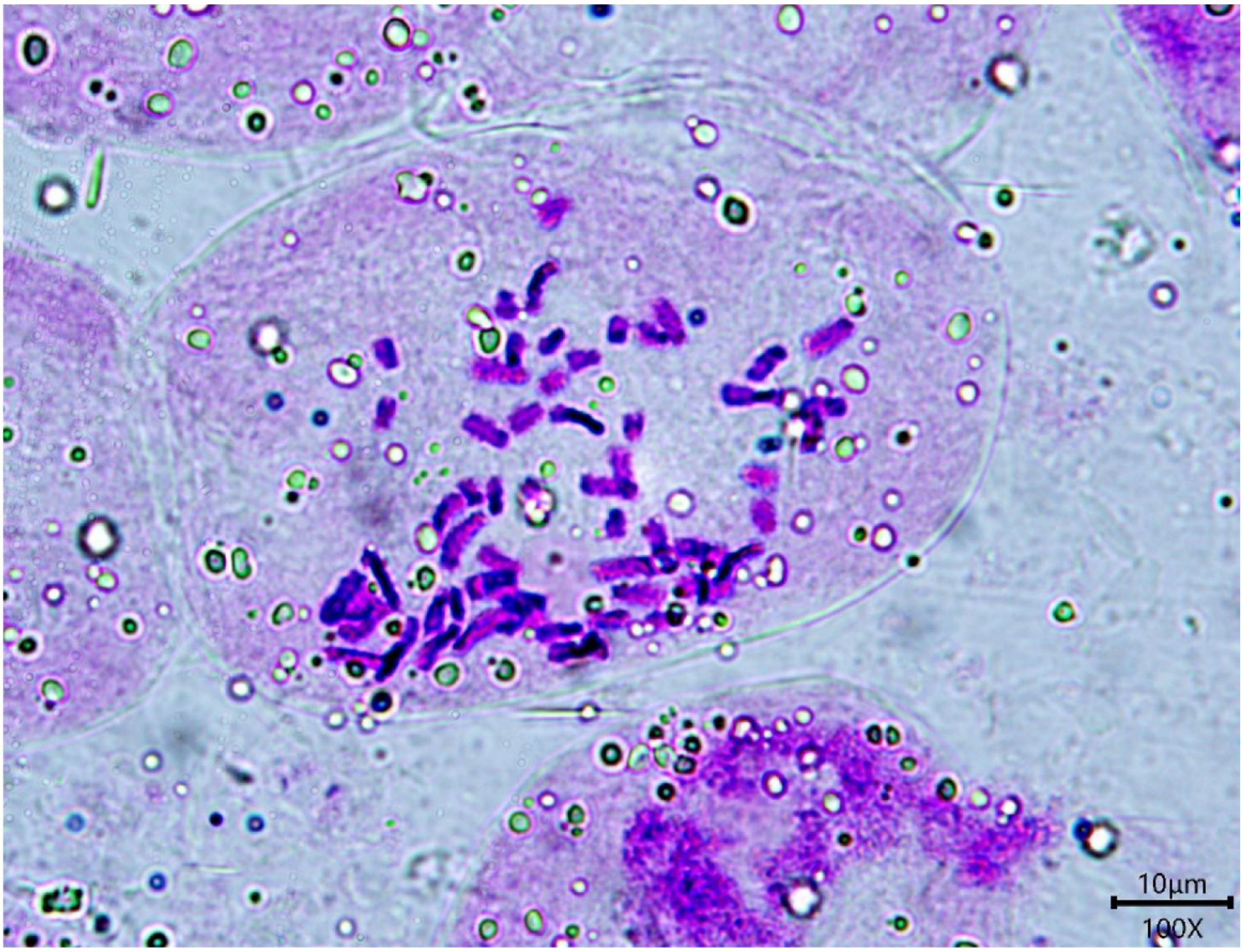

Figure S3. Mitotic chromosomes of *Smilax weniae*. The most probable basic chromosome number seems to be 2*n* = 60.
